# Association between serum uric acid and bone health in general population: a large and multicentre study

**DOI:** 10.18632/oncotarget.6173

**Published:** 2015-10-19

**Authors:** Xianfeng Lin, Chenchen Zhao, An Qin, Dun Hong, Wenyue Liu, Kangmao Huang, Jian Mo, Hejun Yu, Shengjie Wu, Shunwu Fan

**Affiliations:** ^1^ Department of Orthopedics Surgery, Sir Run Run Shaw Hospital, Medical College of Zhejiang University, Hangzhou, China; ^2^ Department of Orthopedics, Shanghai Key Laboratory of Orthopedic Implant, Shanghai Ninth People's Hospital, Shanghai Jiaotong University School of Medicine, Shanghai, China; ^3^ Department of Orthopedics Surgery, Taizhou Hospital of Wenzhou Medical University, Linhai, China; ^4^ Department of Endocrinology, The First Affiliated Hospital of Wenzhou Medical University, Wenzhou, China; ^5^ Department of Cardiology, The Key Laboratory of Cardiovascular Disease of Wenzhou The First Affiliated Hospital of Wenzhou Medical University, Wenzhou, China

**Keywords:** serum uric acid, bone health, bone mineral density, bone mass loss, DXA, Gerotarget

## Abstract

Previous studies proposed that serum uric acid (UA), an endogenous antioxidant, could be a protective factor against bone loss. However, recently, a study with a population of US adults did not note the protective effects of serum UA. Therefore, the exact association between serum UA and bone health remains unclear. We performed a retrospective consecutive cohort study in a Chinese population to examine the association between serum UA and bone health. This cross-sectional study included 17,735 individuals who underwent lumbar spine bone mineral density (BMD) measurements as part of a health examination. In covariance analyses (multivariable-adjusted), a high serum UA level was associated with a high BMD, T-score, and Z-score. In binary logistic regression analyses (multivariable-adjusted), a high serum UA level was associated with low odds ratios (ORs) for at least osteopenia and osteoporosis in male (age ≥50 years) (OR = 0.72–0.60 and OR = 0.49–0.39, respectively) and postmenopausal female participants (OR = 0.61–0.51 and OR = 0.66–0.49, respectively). In conclusion, serum UA is associated with BMD, the T-score, and the Z-score, and has a strong protective effect against at least osteopenia and osteoporosis.

## INTRODUCTION

Uric acid (UA) is the final breakdown product of purine metabolism in humans, and it has been traditionally considered to be a risk factor for hypertension, diabetes mellitus, stroke, cardiovascular disease, renal disease, and metabolic syndrome [1-5]. High amounts of serum UA could lead to the deposition of urate crystals, causing gouty arthritis. However, studies have shown that serum UA might function as an antioxidant against oxidative stress in nervous system diseases such as multiple sclerosis, amyotrophic lateral sclerosis, Alzheimer disease, and dementia [6-9].

Osteoporosis and osteopenia are some of the most common diseases worldwide, and they increase the risk of fractures and are major causes of bone fragility [10, 11]. Many studies have suggested that oxidative stress and low circulating levels of antioxidants are associated with reduced bone mineral density (BMD) and osteoporosis [12-15]. In recent years, studies have assessed whether serum UA is a protective factor against bone loss [16-22]. Since Nabipour et al. first reported that high serum UA levels were associated with high BMD values at all skeletal sites and with low a prevalence of fractures in elderly Australian men (age ≥ 70 years), many studies have shown that high serum UA levels have beneficial effects on bone metabolism in Korean and Japanese perimenopausal and postmenopausal women and in Korean men (age ≥ 50 years) [17-21]. Lane et al. proposed that high serum UA levels were associated with a reduction in the risk of incident non-spine fractures in US men (age ≥ 65 years) [22]. However, the study by Zhang D et al. did not find that high serum UA levels have a protective effect on bone health in US adults (age ≥ 30 years) [16]. Furthermore, their results were confirmed in a rodent model of chronic mild hyperuricemia, demonstrating that the serum UA level was not associated with BMD and bone biomechanical properties.

Therefore, the exact association between serum UA and bone health remains unclear, and a study involving a large multicenter population is required to clarify the association. The present study aimed to investigate the relationship between serum UA and bone loss, including BMD, the T-score, the Z-score, osteoporosis, and osteopenia, in a large multicenter Chinese population.

## RESULTS

### Baseline characteristics

**Figure 1:**
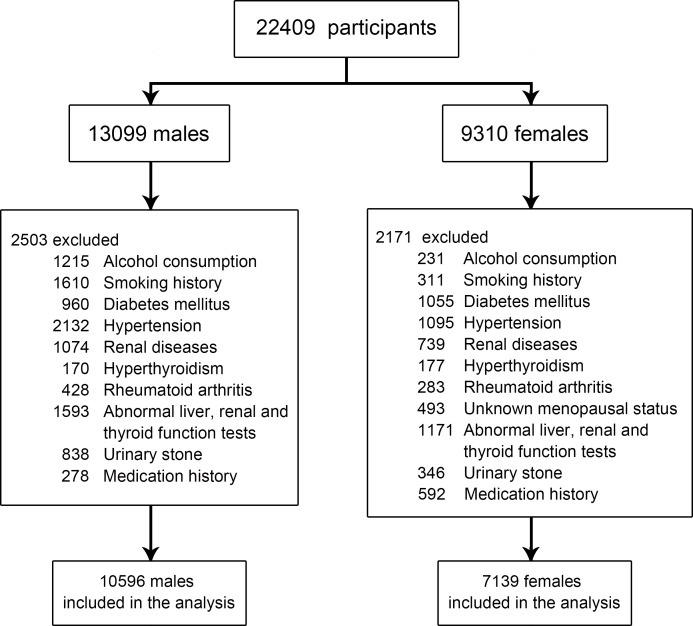
Flow chart of study inclusion A total of 22,409 participants were enrolled. According to the exclusion criteria, 4,674 participants were excluded. Finally, 17,735 participants (10,596 male and 7,139 female participants) were included.

A total of 22,409 participants were enrolled. According to the exclusion criteria, 2,503 male and 2,171 female participants were eliminated. A total of 17,735 participants (10,596 males and 7139 females) with available serum UA and dual-energy X-ray absorptiometry (DXA) measurement data were finally included in this study (Figure 1). The baseline characteristics of the study population are presented in Table 1. The mean serum UA levels in males and females were 374.80 ± 82.76 and 263.79 ± 66.62 mmol/L, respectively. The mean BMD and T-score were higher in male participants than in female participants (1.14 ± 0.15 *vs*. 1.10 ± 0.18, *P* = 0.000 and −0.47 ± 1.36 *vs*. −0.84 ± 1.50, *P* = 0.000, respectively).

**Table 1:** Clinical and biochemical characteristics of the study cohort grouped as males and females

	All participants (*n* = 17735)	Males (*n* = 10596)	Females (*n* = 7139)
UA (mmol/L)	330.57 ± 94.03	374.80 ± 82.76	263.79 ± 66.62
BMD (g/cm2)	1.12 ± 0.17	1.14 ± 0.15	1.10 ± 0.18
T-score	−0.62 ± 1.42	−0.47 ± 1.36	−0.84 ± 1.50
Z-score	0.14 ± 1.34	0.04 ± 1.44	0.29 ± 1.17
Age (years)	49.62 ± 10.46	49.38 ± 11.13	49.98 ± 10.80
Height (cm)	164.06 ± 8.20	168.61 ± 6.29	157.31 ± 5.65
Weight (kg)	65.48 ± 11.30	70.63 ± 10.09	57.84 ± 8.28
BMI (kg/m2)	24.23 ± 3.14	24.81 ± 2.99	23.38 ± 3.16
Systolic BP (mmHg)	123.42 ± 16.31	125.00 ± 15.39	120.81 ± 12.29
Glucose (mmol/L)	5.56 ± 1.41	5.65 ± 1.15	5.44 ± 1.22
Serum calcium (mmol/L)	2.36 ± 0.13	2.37 ± 0.13	2.35 ± 0.13
Serum phosphate(mmol/L)	1.11 ± 0.17	1.08 ± 0.17	1.17 ± 0.16
Alkaline phosphatase (U/L)	74.25 ± 21.85	75.98 ± 19.95	71.63 ± 24.21
AST (U/L)	25.30 ± 18.65	26.79 ± 21.19	23.06 ± 13.65
ALT (U/L)	29.08 ± 36.84	34.13 ± 42.59	21.44 ± 23.88
Triglycerides (mmol/L)	1.78 ± 1.48	2.04 ± 1.64	1.39 ± 1.07
Total cholesterol (mmol/L)	5.08 ± 0.98	5.09 ± 0.97	5.07 ± 0.99
Serum urea nitrogen (mmol/L)	5.16 ± 1.34	5.35 ± 1.31	4.88 ± 1.33
Serum creatinine (μmol/L)	77.85 ± 18.95	85.14 ± 15.22	66.85 ± 18.73
Total bilirubin (μmol/L)	13.67 ± 5.87	14.61 ± 6.25	12.24 ± 4.92
Total protein (g/L)	76.20 ± 4.39	75.96 ± 4.34	76.58 ± 4.44
eGFR (mL/min/1.73m2)	90.41 ± 17.93	90.60 ± 16.13	90.12 ± 20.34

UA, uric acid; BP, blood pressure; AST, aspartate transaminase; ALT, alanine aminotransferase; BMD, bone mineral density; eGFR, estimated glomerular filtration rate.

### Relationship between serum UA levels and BMD

**Figure 2:**
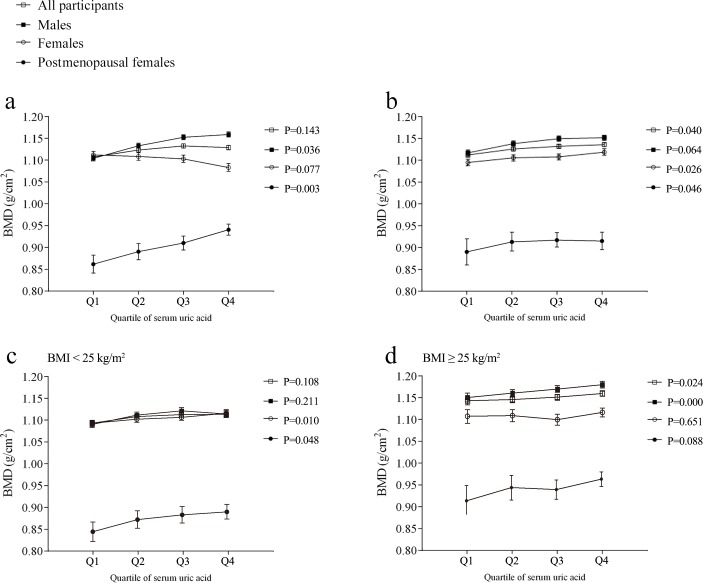
Bone mineral density (BMD) in each quartile of serum uric acid (Q1, Q2, Q3, and Q4) **a.** Unadjusted BMD. **b.** BMD adjusted for age; height; weight; systolic blood pressure; levels of total protein, serum calcium, serum phosphorus, alkaline phosphatase, blood glucose, and blood lipids; and liver function. **c.** Adjusted BMD in the body mass index (BMI) < 25 group. **d.** Adjusted BMD in the BMI ≥ 25 group. Results are presented as the survey weighted least-square means and 95% confidence intervals from regression analysis. *P*-values are from the test for a linear trend across the serum uric acid quartiles.

The participants were divided into four quartiles (Q1, Q2, Q3, and Q4) according to the serum UA levels. Before adjusting for potential confounders, BMD was found to significantly increase across the quartiles in males and postmenopausal females, and a non-significant inverse trend was observed in females (Figure 2a). A covariance analysis was performed to investigate the association between serum UA levels and BMD after controlling for confounders. After adjusting for age; height; weight; systolic blood pressure (BP); levels of serum glucose, total protein, blood urea nitrogen, serum calcium, serum phosphorus, alkaline phosphatase, alanine aminotransferase (ALT), aspartate aminotransferase (AST), triglycerides, total cholesterol, total bilirubin, and serum creatinine; and the estimated glomerular filtration rate (eGFR), BMD was found to have an increasing trend across the quartiles in male, female, and postmenopausal female participants (Figure 2b). BMD and serum UA levels were affected by obesity; therefore, the participants were divided into subgroups according to body mass index (BMI; BMI < 25 and BMI ≥ 25), and the increasing trend was confirmed in the subgroups (Figure 2c and 2d).

### Relationship of serum UA levels with the T-score and Z-score

**Figure 3:**
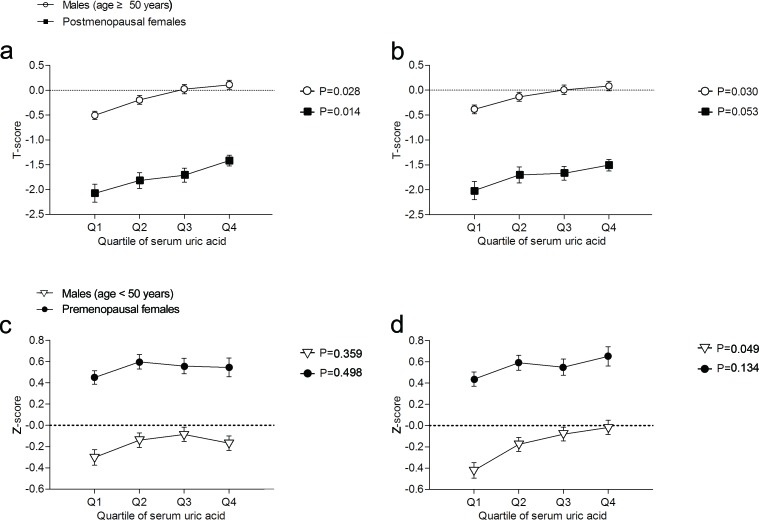
T-score and Z-score in each quartile of serum uric acid (Q1, Q2, Q3, and Q4) in male and female participants **a.** Unadjusted T-score. **b.** T-score adjusted for age; height; weight; systolic blood pressure; levels of total protein, serum calcium, serum phosphorus, alkaline phosphatase, blood glucose, and blood lipids; and liver function. **c.** Unadjusted Z-score. **d.** Z-score adjusted for the abovementioned factors. Results are presented as the survey weighted least-square means and 95% confidence intervals from regression analysis. P-values are from the test for a linear trend across serum uric acid quartiles.

Before adjusting for potential confounders, the T-score was found to significantly increase across the quartiles in males (age ≥ 50 years) and postmenopausal females (Figure 3a). After adjusting for potential confounders, the T-score was found to increase across the quartiles in males and postmenopausal females, although the change in postmenopausal females was not significant (*P* = 0.053) (Figure 3b). Additionally, before adjusting for potential confounders, the Z-score was found to have no remarkable increasing trend across the quartiles in male (age < 50 years) and premenopausal female participants (Figure 3c). However, after adjusting for potential confounders, the Z-score was found to significantly increase across the quartiles in males (age < 50 years) (*P* = 0.049; Figure 3d).

### Prevalence of at least osteopenia and osteoporosis in the serum UA quartiles

**Figure 4:**
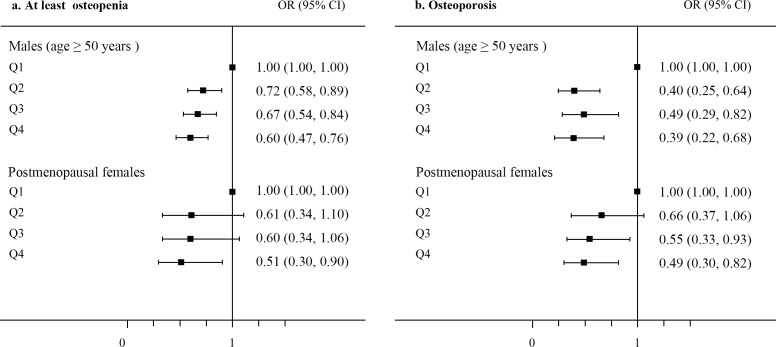
**Odds ratios (ORs) and 95% confidence intervals (CI) for a.** at least osteopenia and **b.** osteoporosis according to the serum uric acid quartiles after adjusting for confounders.

Univariate analysis showed that the odds ratios (ORs) for both at least osteopenia and osteoporosis decreased significantly from Q1 to Q4 (Supplemental Tables 1 and 2). Additionally, after adjusting for potential confounders, the multivariate analysis revealed that the ORs for both at least osteopenia and osteoporosis decreased significantly from Q1 to Q4 in males (age ≥ 50 years) in a dose-dependent manner (Figure 4a and Supplemental Table 3). Among the male participants, compared to the OR for at least osteopenia in Q1, the ORs were 40% lower in Q4, 33% lower in Q3, and 28% lower in Q2. The ORs and 95% confidence intervals (CI) for osteoporosis among the male participants in Q2, Q3, and Q4 were 0.395 (0.245-0.637), 0.491 (0.295-0.818), and 0.386 (0.220-0.678), respectively (Figure 4b and Supplemental Table 3). Additionally, the ORs and 95% CI for at least osteopenia and osteoporosis among the postmenopausal female participants in Q4 were 0.515 (0.299-0.889) and 0.494 (0.299-0.818), respectively (Figure 4a and 4b, Supplemental Table 4). Moreover, the ORs and 95% CI for osteoporosis among the postmenopausal female participants in Q2 and Q3 were 0.662 (0.366-1.058) and 0.551 (0.328-0.928), respectively (Figure 4b, Supplemental Table 4), which are consistent with the above results. A stratified analysis for obesity (BMI < 25 and BMI ≥ 25) showed a successive decrease in ORs in males (age ≥ 50 years) and postmenopausal females for at least osteopenia and osteoporosis (Supplemental Figure 1).

## DISCUSSION

The present study found that a high serum UA level was associated with a high BMD, T-score, and Z-score, and low ORs for at least osteopenia and osteoporosis.

Previous studies speculated that serum UA could serve as an antioxidant in patients with osteoporosis and could prevent the reduction of BMD in certain populations [17-21]. Recently, a study by Zhang D et al. did not show the protective effects of UA in a general US population, and the negative results were confirmed in an animal study [16]. The majority of these previous studies mainly focused on the association between serum UA levels and BMD, and ignored other related variables, such as the T-score, Z-score, and prevalence of osteoporosis or osteopenia. The T-score and Z-score are considered key variables in the evaluation of bone loss.

In the present study, we found a positive association between serum UA levels and BMD after adjusting for confounders, which differs from the finding of the study performed by Zhang D et al. [16]. Additionally, further analysis demonstrated a positive interrelated relationship of serum UA levels with the T-score and Z-score. Moreover, the ORs for at least osteopenia and osteoporosis decreased from the low serum UA group to the high serum UA group in a dose-dependent manner. Furthermore, the protective effect of serum UA on bone health was confirmed in normal weight and overweight participants. These findings indicated that the trends were highly consistent independent of BMD, the T-score, the Z-score, at least osteopenia, and osteoporosis.

We comprehensively analyzed BMD, the T-score, the Z-score, and ORs for at least osteopenia and osteoporosis in this study. As with BMD, the T-score and Z-score might accurately and directly reflect the bone loss of specific individuals by considering age, sex, and ethnicity. Additionally, T-score-based subgroup (ORs for at least osteopenia and osteoporosis) analyses might help to amplify the minor differences in bone loss among different serum UA levels. Our study participants were recruited from five medical centers in China, and this might attenuate the population and regional variances in the present study.

Serum UA is a commonly measured biochemical parameter in health examinations and our results provide epidemiological evidence that serum UA might have a beneficial effect on bone health. Therefore, serum UA might be used to select individuals for DXA in order to assess osteoporosis or osteopenia.

The present study has several strengths. First, we collected data from 17,735 participants at five medical centers in China. The data included a broad spectrum of anthropometrics, laboratory assays and measurements, and BMD measurement variables. Additionally, the present study included participants with a wide age range (20-90 years). Second, we performed a comprehensive analysis of the correlation between serum UA levels and bone loss. Furthermore, a dose-dependent protective effect of serum UA on bone loss was noted in our study.

The present study has some limitations. First, this was a cross-sectional study; therefore, it is difficult to conclude that there is a causal relationship between serum UA levels and bone loss. The follow-up of populations is important and meaningful. Second, fractures are one of the most common and serious complications of bone loss, and their incidence should be recorded in a further study. Third, this study did not evaluate dietary intake, such as intake of vegetables and fruits, which might have influenced bone health.

This large and multicenter general population-based study found that serum UA is associated with BMD, the T-score, and the Z-score, and has a strong protective effect against at least osteopenia and osteoporosis. Further studies are needed to elucidate the exact mechanisms by which serum UA contributes to bone loss.

## MATERIALS AND METHODS

### Study population

We performed a retrospective and consecutive cohort study from October 2009 to December 2014 at the following five medical centers: Sir Run Run Shaw Hospital Affiliated to Zhejiang University, Shanghai Ninth People's Hospital Affiliated to Shanghai Jiaotong University School of Medicine, First Affiliated Hospital of Wenzhou Medical University, Taizhou Enze Medical Center, and Shaoxing People's Hospital. The study participants consisted entirely of community individuals who visited these medical centers for health examinations and underwent extensive screening tests for the early detection of diseases, such as malignancy and osteoporosis. A total of 22,409 participants were enrolled. Participants diagnosed with diabetes mellitus, hypertension, hyperthyroidism, rheumatoid arthritis, gout, or urinary stones were excluded. Additionally, participants with a history of alcohol consumption (more than 20 g alcohol per day with current or past) or smoking (more than 10 cigarettes per day with current or past) and women with unknown menopausal status (MS) were excluded. Moreover, participants with abnormal results in liver, renal, or thyroid function tests were excluded from this study. Furthermore, participants on medications that could influence bone metabolism, such as diuretics, estrogens, and bisphosphonates, for a long period ( > 3 months) were excluded. This study was approved by the ethics committees of all five medical centers, and the requirement of informed consent was waived owing to the retrospective nature of the study.

### Participant baseline characteristics

Data on the baseline characteristics of the participant were collected. Age, smoking history, alcohol consumption history, medication history, MS, and medical history were recorded using a standardized questionnaire. MS was categorized as follows: 1, premenopausal (regular menstrual cycles); 2, perimenopausal (menses or amenorrhea of at least three months but less than 12 months); and 3, postmenopausal (amenorrhea for 12 consecutive months). BP was measured in the resting state with a standard mercury sphygmomanometer. Height and weight were measured by trained nurses, with participants wearing a light gown, and BMI was calculated for all participants.

### BMD measurements

Areal BMD (g/cm^2^) of the lumbar spine (L1 to L4) was measured using DXA with Lunar equipment (Prodigy; Lunar, Madison, WI). All measurements were taken by experienced operators on the same parameter settings following standardized procedures. A standard quality control (QC) program, including daily calibrations with machine-specific phantoms, was used in the medical centers. A total of five Lunar devices were used in this study, and they were cross-calibrated. The same densitometer was used throughout the study. The performance of the DXA scanner was monitored throughout the study. Routine daily QC scans of a spine phantom were performed, and the coefficients of variation (CVs) for QC BMD measurements in the five medical centers were 0.91%, 0.85%, 0.82%, 0.95%, and 0.89%. *In vivo* reproducibilities were estimated from duplicate scans (60 patients with repositioning between scans in each medical center) as the CVs for BMD. The CVs for lumbar spine BMD in the five medical centers were 1.15%, 1.07%, 0.95%, 1.22%, and 1.01%. The BMD measurements provided absolute values for the lumbar spine, and the BMD values were compared with those of healthy young Chinese adults of the same sex and ethnicity (T-score) or an age-, sex-, and ethnicity-matched reference population (Z-score). According to the World Health Organization, the T-score is the relevant measure when screening for osteoporosis and osteopenia in postmenopausal women and men aged ≥ 50 years. The classification criteria of the World Health Organization are as follows: normal (T-score ≥ −1.0), osteopenia (−1.0 > T-score > −2.5), and osteoporosis (T-score ≤ −2.5). In this study, participants with osteoporosis and osteopenia were classified as “at least osteopenia.”

### Laboratory assays and measurements

During the health examinations, routine blood biochemical tests, including serum UA, total protein, blood urea nitrogen, serum calcium, serum phosphorus, alkaline phosphatase, liver function, blood glucose, and blood lipids, were performed. The liver function tests included ALT, AST, and total bilirubin. The blood lipid tests included total cholesterol, low-density lipoprotein cholesterol, high-density lipoprotein cholesterol, and triglycerides. The eGFR was calculated according to age, sex, race/ethnicity, and the serum creatinine level using the Chronic Kidney Disease Epidemiology Collaboration (CKD-EPI) equation, which has been shown to perform well in the Chinese population [23, 24]. The CKD-EPI equation is as follows: GFR (mL/min/1.73 m^2^) = 141 × min (Scr/κ, 1)^α^ × max (Scr/κ, 1) ^−1.209^ × 0.993^Age^ × 1.018 [if female] × 1.159 [if black] (κ: male, 0.9; female, 0.7 and α: male, −0.411; female, −0.329). The participants were divided into quartiles according to the serum UA levels as follows: Q1: ≤ 320 mmol/L, Q2: 321-370 mmol/L, Q3: 371-425 mmol/L, and Q4 ≥ 426 mmol/L for males, and Q1 ≤ 220 mmol/L, Q2: 221-260 mmol/L, Q3: 261-300 mmol/L, and Q4 ≥ 301 mmol/L for females.

### Statistical analysis

The data are presented as means ± standard deviations or percentages. Categorical variables were compared using the χ^2^ test or Fisher's exact test, as appropriate, and continuous variables were compared using the Wilcoxon signed rank test. The relationships of serum UA levels with BMD, the T-score, the Z-score, osteopenia, and osteoporosis were investigated after controlling for various confounders. The potential confounding factors included age; height; weight; systolic BP; levels of total protein, serum calcium, serum phosphorus, alkaline phosphatase, blood glucose, and blood lipids; and liver function. Linear regression analyses and covariance analyses were performed to estimate the trends of the mean BMD, T-score, and Z-score across the increasing subgroup-specific quartiles of serum UA in unadjusted and multivariable-adjusted models. Binary logistic regression analyses were performed to determine the correlation of serum UA levels with osteoporosis and at least osteopenia after adjusting for potential confounders. ORs and corresponding 95% CI were calculated. All statistical analyses were performed using SPSS 16.0 software (IBM, Armonk, NY). A two-sided *P*-value < 0.05 was considered statistically significant.

## SUPPLEMENTARY MATERIAL FIGURE AND TABLES



## References

[1] Johnson RJ, Kang DH, Feig D, Kivlighn S, Kanellis J, Watanabe S, Tuttle KR, Rodriguez-Iturbe B, Herrera-Acosta J, Mazzali M (2003). Is there a pathogenetic role for uric acid in hypertension and cardiovascular and renal disease?. Hypertension.

[2] Dehghan A, van Hoek M, Sijbrands EJ, Hofman A, Witteman JC (2008). High serum uric acid as a novel risk factor for type 2 diabetes. Diabetes Care.

[3] Holme I, Aastveit AH, Hammar N, Jungner I, Walldius G (2009). Uric acid and risk of myocardial infarction, stroke and congestive heart failure in 417,734 men and women in the Apolipoprotein MOrtality RISk study (AMORIS). J Intern Med.

[4] Verdecchia P, Schillaci G, Reboldi G, Santeusanio F, Porcellati C, Brunetti P (2000). Relation between serum uric acid and risk of cardiovascular disease in essential hypertension. The PIUMA study. Hypertension.

[5] Bergamini C, Cicoira M, Rossi A, Vassanelli C (2009). Oxidative stress and hyperuricaemia: pathophysiology, clinical relevance, and therapeutic implications in chronic heart failure. Eur J Heart Fail.

[6] Massa J, O'Reilly E, Munger KL, Delorenze GN, Ascherio A (2009). Serum uric acid and risk of multiple sclerosis. J Neurol.

[7] Keizman D, Ish-Shalom M, Berliner S, Maimon N, Vered Y, Artamonov I, Tsehori J, Nefussy B, Drory VE (2009). Low uric acid levels in serum of patients with ALS: further evidence for oxidative stress?. J Neurol Sci.

[8] Andreadou E, Nikolaou C, Gournaras F, Rentzos M, Boufidou F, Tsoutsou A, Zournas C, Zissimopoulos V, Vassilopoulos D (2009). Serum uric acid levels in patients with Parkinson's disease: their relationship to treatment and disease duration. Clin Neurol Neurosurg.

[9] Euser SM, Hofman A, Westendorp RG, Breteler MM (2009). Serum uric acid and cognitive function and dementia. Brain.

[10] Manolagas SC (2010). From estrogen-centric to aging and oxidative stress: a revised perspective of the pathogenesis of osteoporosis. Endocr Rev.

[11] Varacallo MA, Fox EJ (2014). Osteoporosis and its complications. Med Clin North Am.

[12] Maggio D, Barabani M, Pierandrei M, Polidori MC, Catani M, Mecocci P, Senin U, Pacifici R, Cherubini A (2003). Marked decrease in plasma antioxidants in aged osteoporotic women: results of a cross-sectional study. J Clin Endocrinol Metab.

[13] Sugiura M, Nakamura M, Ogawa K, Ikoma Y, Ando F, Yano M (2008). Bone mineral density in post-menopausal female subjects is associated with serum antioxidant carotenoids. Osteoporos Int.

[14] Sugiura M, Nakamura M, Ogawa K, Ikoma Y, Ando F, Shimokata H, Yano M (2011). Dietary patterns of antioxidant vitamin and carotenoid intake associated with bone mineral density: findings from post-menopausal Japanese female subjects. Osteoporos Int.

[15] Sendur OF, Turan Y, Tastaban E, Serter M (2009). Antioxidant status in patients with osteoporosis: a controlled study. Joint Bone Spine.

[16] Zhang D, Bobulescu IA, Maalouf NM, Adams-Huet B, Poindexter J, Park S, Wei F, Chen C, Moe OW, Sakhaee K (2015). Relationship Between Serum Uric Acid and Bone Mineral Density in the General Population and in Rats with Experimental Hyperuricemia. J Bone Miner Res.

[17] Nabipour I, Sambrook PN, Blyth FM, Janu MR, Waite LM, Naganathan V, Handelsman DJ, Le Couteur DG, Cumming RG, Seibel MJ (2011). Serum uric acid is associated with bone health in older men: a cross-sectional population-based study. J Bone Miner Res.

[18] Ahn SH, Lee SH, Kim BJ, Lim KH, Bae SJ, Kim EH, Kim HK, Choe JW, Koh JM, Kim GS (2013). Higher serum uric acid is associated with higher bone mass, lower bone turnover, and lower prevalence of vertebral fracture in healthy postmenopausal women. Osteoporos Int.

[19] Makovey J, Macara M, Chen JS, Hayward CS, March L, Seibel MJ, Sambrook PN (2013). Serum uric acid plays a protective role for bone loss in peri- and postmenopausal women: a longitudinal study. Bone.

[20] Ishii S, Miyao M, Mizuno Y, Tanaka-Ishikawa M, Akishita M, Ouchi Y (2014). Association between serum uric acid and lumbar spine bone mineral density in peri- and postmenopausal Japanese women. Osteoporos Int.

[21] Kim BJ, Baek S, Ahn SH, Kim SH, Jo MW, Bae SJ, Kim HK, Choe J, Park GM, Kim YH, Lee SH, Kim GS, Koh JM (2014). Higher serum uric acid as a protective factor against incident osteoporotic fractures in Korean men: a longitudinal study using the National Claim Registry. Osteoporos Int.

[22] Lane NE, Parimi N, Lui LY, Wise BL, Yao W, Lay YA, Cawthon PM, Orwoll E (2014). Association of serum uric acid and incident nonspine fractures in elderly men: the Osteoporotic Fractures in Men (MrOS) study. J Bone Miner Res.

[23] Levey AS, Stevens LA, Schmid CH, Zhang YL, Castro AF, Feldman HI, Kusek JW, Eggers P, Van Lente F, Greene T, Coresh J (2009). A new equation to estimate glomerular filtration rate. Ann Intern Med.

[24] Kong X, Ma Y, Chen J, Luo Q, Yu X, Li Y, Xu J, Huang S, Wang L, Huang W, Wang M, Xu G, Zhang L, Zuo L, Wang H (2013). Evaluation of the Chronic Kidney Disease Epidemiology Collaboration equation for estimating glomerular filtration rate in the Chinese population. Nephrol Dial Transplant.

