# Supplementary material for: Association between serum uric acid and bone health in general population: a large and multicentre study
Source: Oncotarget. 2015 Oct 19;6(34):35395–403. doi: 10.18632/oncotarget.6173 (PMC4742113; doi:10.18632/oncotarget.6173)
Supplement: Supplementary file 1 [file oncotarget-06-35395-s001.pdf]

Association between serum uric acid and bone health in general population: a large and multicentre study

Supplementary Material

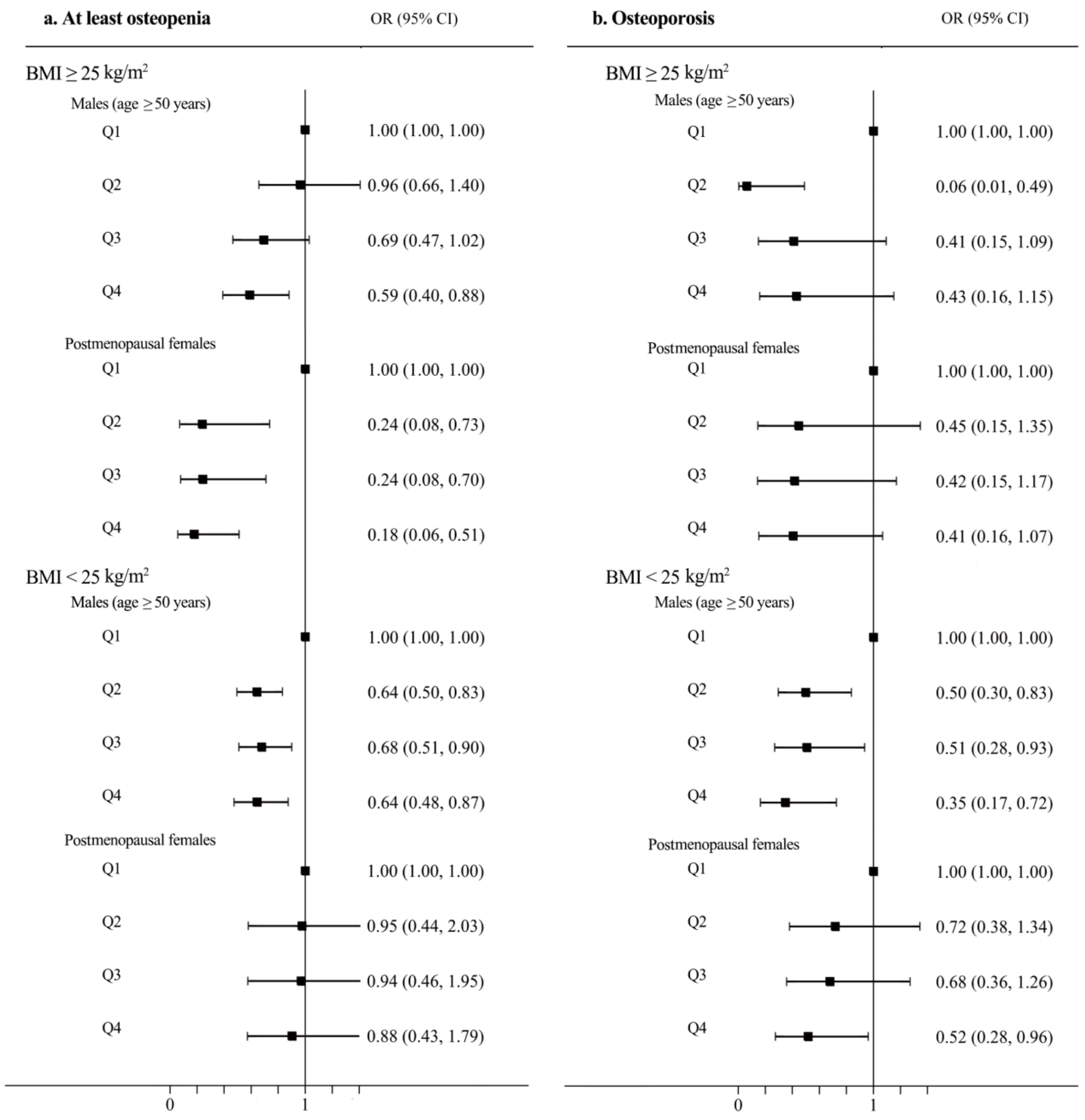

Supplemental Figure 1: Odds ratios (ORs) and 95% confidence intervals (CI) for a, b) at least osteopenia and c, d) osteoporosis in body mass index (BMI) subgroups (BMI < 25 and BMI ≥ 25) according to the serum uric acid quartiles after adjusting for confounders.

Supplemental table 1. Univariate analysis of the risk for at least osteopenia and osteoporosis in males with age more than 50 years

	At least osteopenia		Osteoporosis	
	OR (95% CI)	P-value	OR (95% CI)	P-value
Q2 (mmol/L)	**0.650 (0.547 - 0.773)**	**0.000**	**0.502 (0.344 - 0.732)**	**0.000**
Q3 (mmol/L)	**0.527 (0.438 - 0.632)**	**0.000**	**0.428 (0.284 - 0.646)**	**0.000**
Q4 (mmol/L)	**0.510 (0.425 - 0.613)**	**0.000**	**0.331 (0.211 - 0.519)**	**0.000**
Age (years)	1.024 (1.016 - 1.032)	0.000	1.048 (1.033 - 1.064)	0.000
Weight (kg)	0.944 (0.937 - 0.951)	0.000	0.925 (0.910 - 0.940)	0.000
Height (cm)	0.971 (0.961 - 0.981)	0.000	0.946 (0.925 - 0.967)	0.000
Systolic BP (mmHg)	0.990 (0.985 - 0.994)	0.000	0.987 (0.977 - 0.996)	0.006
Glucose (mmol/L)	0.936 (0.894 - 0.981)	0.005	0.884 (0.775 - 1.009)	0.067
Serum calcium (mmol/L)	0.758 (0.433 - 1.326)	0.332	0.534 (0.162 - 1.762)	0.303
Serum phosphorus (mmol/L)	1.013 (0.647 - 1.586)	0.956	1.481 (0.537 - 4.086)	0.449
Alkaline phosphatase (U/L)	1.012 (1.009 - 1.016)	0.000	1.015 (1.009 - 1.021)	0.000
AST (U/L)	0.998 (0.994 - 1.002)	0.386	1.001 (0.997- 1.005)	0.736
ALT (U/L)	0.993 (0.989 - 0.997)	0.000	0.987 (0.976 - 0.997)	0.015
Triglycerides (mmol/L)	0.920 (0.873 - 0.970)	0.002	0.791 (0.675 - 0.928)	0.004
Total cholesterol (mmol/L)	1.080 (1.010 - 1.155)	0.024	1.108 (0.957- 1.283)	0.170
Serum urea nitrogen (mmol/L)	0.959 (0.916 - 1.004)	0.074	0.959 (0.864- 1.064)	0.427
Serum creatinine (µmol/L)	1.011 (1.007 - 1.015)	0.000	1.016 (1.008 - 1.024)	0.000
Total bilirubin (µmol/L)	1.002 (0.992 - 1.012)	0.669	1.010 (0.993 - 1.029)	0.254
Total protein (g/L)	1.002 (0.988 - 1.017)	0.742	1.007 (0.974 - 1.040)	0.689
eGFR (mL/min/1.73m^2)	0.984 (0.980 - 0.988)	0.000	0.974 (0.965 - 0.983)	0.000

BP, blood pressure; AST, aspartate transaminase; ALT, alanine aminotransferase; BMD, bone mineral density; eGFR, estimated glomerular filtration rate; OR, odds ratio; CI, confidence intervals.

The reference group was Q1.

Supplemental table 2. Univariate analysis of the risk for at least osteopenia and osteoporosis in postmenopausal females

	At least osteopenia		Osteoporosis	
	OR (95% CI)	P-value	OR (95% CI)	P-value
Q2 (mmol/L)	**0.707 (0.433 - 1.153)**	**0.164**	**0.706 (0.470 - 1.062)**	**0.095**
Q3 (mmol/L)	**0.604(0.380 - 0.959)**	**0.033**	**0.553 (0.372 - 0.821)**	**0.003**
Q4 (mmol/L)	**0.417 (0.272 - 0.639)**	**0.000**	**0.406 (0.279 - 0.589)**	**0.000**
Age (years)	1.017 (0.996 - 1.039)	0.115	1.047 (1.026 - 1.068)	0.000
Weight (kg)	0.934 (0.920 - 0.948)	0.000	0.908 (0.891 - 0.925)	0.000
Height (cm)	0.929 (0.907 - 0.951)	0.000	0.918 (0.897 - 0.941)	0.000
Systolic BP (mmHg)	0.992 (0.985 - 0.999)	0.024	0.991 (0.984 - 0.999)	0.021
Glucose (mmol/L)	0.956 (0.883 - 1.035)	0.270	0.821 (0.727 - 0.927)	0.001
Serum calcium (mmol/L)	1.047 (0.356 - 3.077)	0.933	0.553 (0.189 - 1.619)	0.280
Serum phosphorus (mmol/L)	0.688 (0.286 - 1.655)	0.404	0.980 (0.397 – 2.422)	0.966
Alkaline phosphatase (U/L)	1.013 (1.008 -1.019)	0.000	1.013 (1.008 - 1.018)	0.000
AST (U/L)	1.004 (0.993 - 1.017)	0.462	1.007 (0.996 - 1.018)	0.213
ALT (U/L)	0.998 (0.990 - 1.005)	0.498	0.997 (0.989 - 1.005)	0.433
Triglycerides (mmol/L)	0.895 (0.797 - 1.005)	0.061	0.842 (0.730 - 0.972)	0.019
Total cholesterol (mmol/L)	1.060 (0.935 - 1. 201)	0.360	1.082 (0.955 - 1.226)	0.216
Serum urea nitrogen (mmol/L)	0.998 (0.917 - 1.086)	0.963	1.012 (0.928 - 1.103)	0.787
Serum creatinine (μmol/L)	0.995 (0.989 - 1.001)	0.112	0.997 (0.991 - 1.004)	0.437
Total bilirubin (μmol/L)	1.010 (0.985 - 1.035)	0.447	0.990 (0.965 - 1.016)	0.444
Total protein (g/L)	0.992 (0.966 - 1.020)	0.587	0.990 (0.962 - 1.018)	0.479
eGFR (mL/min/1.73m^2)	1.005 (0.998 - 1.011)	0.163	1.001 (0.994 - 1.007)	0.859

Abbreviations as in supplemental table 1. The reference group was Q1.

Supplemental table 3. Multivariate analysis of the risk for at least osteopenia and osteoporosis in males with age more than 50 years

	At least osteopenia		Osteoporosis	
	OR (95% CI)	P-value	OR (95% CI)	P-value
Q2 (mmol/L)	**0.720 (0.585 - 0.887)**	**0.002**	**0.395 (0.245 - 0.637)**	**0.000**
Q3 (mmol/L)	**0.670 (0.535 - 0.837)**	**0.000**	**0.491 (0.295 - 0.818)**	**0.006**
Q4 (mmol/L)	**0.599 (0.473 - 0.759)**	**0.000**	**0.386 (0.220 - 0.678)**	**0.001**
Age (years)	0.962 (0.939 - 0.985)	0.002	0.926 (0.901 - 0.951)	0.158
Weight (kg)	0.932 (0.921 - 0.943)	0.000	0.926 (0.901 - 0.951)	0.000
Height (cm)	1.023 (1.007 - 1.039)	0.004	1.013 (0.979 - 1.048)	0.460
Systolic BP (mmHg)	0.996 (0.990 - 1.001)	0.099	0.999 (0.987 - 1.010)	0.822
Glucose (mmol/L)	0.946 (0.900 - 0.994)	0.029	0.946 (0.847 - 1.057)	0.326
Serum calcium (mmol/L)	0.638 (0.320 - 1.274)	0.203	0.559 (0.131 - 2.739)	0.509
Serum phosphorus (mmol/L)	1.614 (0.957 - 2.724)	0.073	3.004 (1.016 - 8.881)	0.047
Alkaline phosphatase (U/L)	1.016 (1.012 - 1.020)	0.000	1.014 (1.007 - 1.021)	0.000
AST (U/L)	0.989 (0.979 - 0.999)	0.040	1.004 (0.986 - 1.022)	0.702
ALT (U/L)	1.002 (0.995 - 1.008)	0.628	0.991 (0.976 - 1.006)	0.250
Triglycerides (mmol/L)	0.994 (0.933 - 1.060)	0.861	0.936 (0.785 - 1.116)	0.462
Total cholesterol (mmol/L)	1.175 (1.077 - 1.282)	0.000	1.232 (1.024 - 1.483)	0.027
Serum urea nitrogen (mmol/L)	0.911 (0.860 - 0.965)	0.002	0.890 (0.783 - 1.011)	0.073
Serum creatinine (µmol/L)	0.933 (0.902 - 0.964)	0.000	0.929 (0.856 - 1.009)	0.080
Total bilirubin (µmol/L)	0.998 (0.985 - 1.011)	0.766	1.005 (0.982 - 1.029)	0.670
Total protein (g/L)	1.000 (0.981 - 1.020)	0.973	1.009 (0.968 - 1.051)	0.668
eGFR (mL/min/1.73m^2)	0.905 (0.872 - 0.940)	0.000	0.894 (0.815 - 0.980)	0.017

Abbreviations as in supplemental table 1. The reference group was Q1.

Supplemental table 4. Multivariate analysis of the risk for at least osteopenia and osteoporosis in postmenopausal females

	At least osteopenia		Osteoporosis	
	OR (95% CI)	P-value	OR (95% CI)	P-value
Q2 (mmol/L)	**0.608 (0.335 - 1.101)**	**0.100**	**0.662 (0.366 - 1.058)**	**0.080**
Q3 (mmol/L)	**0.603 (0.343 - 1.059)**	**0.078**	**0.551 (0.328 - 0.928)**	**0.025**
Q4 (mmol/L)	**0.515 (0.299 - 0.889)**	**0.017**	**0.494 (0.299 - 0.818)**	**0.006**
Age (years)	1.020 (0.980 - 1.062)	0.331	1.077 (1.031 - 1.125)	0.001
Weight (kg)	0.943 (0.923 - 0.963)	0.000	0.924 (0.900 - 0.948)	0.000
Height (cm)	0.971 (0.939 - 1.003)	0.079	0.953 (0.919 - 0.988)	0.009
Systolic BP (mmHg)	0.996 (0.987 - 1.006)	0.421	0.993 (0.982 - 1.003)	0.153
Glucose (mmol/L)	0.986 (0.897 - 1.085)	0.774	0.834 (0.723 - 0.961)	0.012
Serum calcium (mmol/L)	1.328 (0.369 - 4.777)	0.664	0.499 (0.116 - 2.143)	0.350
Serum phosphorus (mmol/L)	0.625 (0.224 - 1.747)	0.370	1.352 (0.433 - 4.224)	0.604
Alkaline phosphatase (U/L)	1.013 (1.005 -1.020)	0.001	1.013 (1.006 - 1.020)	0.000
AST (U/L)	1.033 (1.003 - 1.065)	0.032	1.015 (0.984 - 1.048)	0.339
ALT (U/L)	0.986 (0.969 - 1.003)	0.108	0.995 (0.975 - 1.015)	0.591
Triglycerides (mmol/L)	1.056 (0.896 - 1.245)	0.516	1.011 (0.847 - 1.207)	0.904
Total cholesterol (mmol/L)	1.098 (0.929 - 1. 298)	0.271	1.171 (0.978 - 1.403)	0.085
Serum urea nitrogen (mmol/L)	1.030 (0.916 - 1.158)	0.620	1.018 (0.900 - 1.151)	0.776
Serum creatinine (μmol/L)	0.984 (0.935 - 1.036)	0.544	1.013 (0.957 - 1.071)	0.665
Total bilirubin (μmol/L)	1.007 (0.975 - 1.039)	0.682	0.981 (0.948 - 1.016)	0.290
Total protein (g/L)	0.984 (0.946 - 1.023)	0.404	1.013 (0.970 - 1.057)	0.564
eGFR (mL/min/1.73m^2)	0.996 (0.947 - 1.048)	0.873	1.025 (0.969 - 1.085)	0.390

Abbreviations as in supplemental table 1. The reference group was Q1.
